# *N-*glycosylation alterations in expressed prostatic secretions: exploring liquid biopsy biomarkers for prostate cancer diagnosis and disease progression

**DOI:** 10.7717/peerj.21401

**Published:** 2026-06-09

**Authors:** Ivy Yee Yen Chew, Jaime Jacqueline Jayapalan, Jasmine Lim

**Affiliations:** 1 Department of Surgery, Faculty of Medicine, Universiti Malaya, Kuala Lumpur, Wilayah Persekutuan, Malaysia; 2 Department of Molecular Medicine, Faculty of Medicine, Universiti Malaya, Kuala Lumpur, Wilayah Persekutuan, Malaysia

**Keywords:** Prostate cancer, Glycosylation, Glycomics, Expressed prostatic secretions (EPS), Extracellular vesicles (EV), Liquid biopsy, Biomarkers

## Abstract

Prostate cancer (PCa) is among the most common malignancies in men. Liquid biopsies enable repeated sampling of tumor-derived material, including extracellular vesicles (EVs) that provide stable, multianalyte molecular readouts. Expressed prostatic secretion (EPS), obtained directly or as EPS-urine after prostate massage, is an organ-proximal matrix enriched in prostate-derived proteins, metabolites, and prostasome-like EVs, but its use is limited by low volumes, urine dilution, and lack of standardized protocols. Glycosylation, is a non-templated post-translational modification regulated by enzyme networks and nucleotide-sugar availability, allowing glycomic phenotypes to capture disease states beyond gene or protein levels. This review covers studies from 1985 to 2025 on EPS-associated glycosylation in PCa and, despite limited and heterogenous data, consistently reports altered core fucosylation, terminal sialylation, and *N-*glycan branching. EV-associated glycomes and prostate-specific antigen glycoforms may diverge from bulk EPS glycans, reflecting selective vesicular cargo loading and compartment-specific regulation. Integrating EPS-based glycan profiling with existing tools could enhance PCa diagnosis and longitudinal monitoring. This review synthesizes emerging findings on EPS-derived glycomic alterations in PCa and presents EPS as a promising yet underutilized biofluid for non-invasive biomarker discovery. Nevertheless, rigorous validation through large-scale studies is essential to establish EPS-based glycan biomarkers as reliable diagnostic and prognostic tools for PCa.

## Introduction

Prostate cancer (PCa) is the fourth most commonly diagnosed cancer worldwide and the eighth leading cause of cancer mortality, with an estimated 1.47 million new cases and 397,430 deaths in 2020 (Bray et al., 2024). In Malaysia, PCa is the third most common cancer among men, with cases rising from 1,682 (2012–2016) to 2,655 (2017–2021). The lifetime risk is about 1 in 83, and 67% of PCa cases are diagnosed at Stage III and IV (Lim et al., 2021; National Cancer Registry, 2023). Established risk factors for PCa include increasing age, ethnicity, family history and environmental influences (Sekhoacha et al., 2022).

PCa diagnosis relies on digital rectal examination (DRE), serum prostate-specific antigen (PSA), imaging and prostate biopsy (Heidenreich et al., 2008). The currently used clinical diagnostic tools for PCa are summarized in Table 1. PSA testing remains central for identifying men who require further evaluation, typically with multiparametric magnetic resonance imaging (MRI) and targeted or systematic biopsy (Merriel et al., 2022). Biopsy remains the final and definitive step in confirming a clinically significant PCa diagnosis (Heidenreich et al., 2008). Quantitative imaging approaches such as radiomics link MRI or biopsy features with tumor aggressiveness, treatment response, and prognosis, and expressed prostatic secretion (EPS)-derived molecular profiles could complement these image-based signatures in integrated risk models for PCa (Tapper et al., 2024). Once a diagnosis is established, clinicians must determine whether active treatment is necessary, and active surveillance strategies with serial PSA, DRE, and repeat imaging/biopsy are commonly used (De Vos, Luiting & Roobol, 2023).

**Table 1:** Currently used clinical diagnostic tools for PCa with procedures, strengths, limitations, and diagnostic performance.

**Diagnostic tool**	**Procedure**	**Strengths**	**Weaknesses**	**Diagnostic performance**		**Invasiveness**	**References**
				**Sensitivity**	**Specificity**		
DRE	Palpation of the prostate gland *via* the rectum using a gloved, lubricated finger	i. Cost-effective ii. No specialized equipment required iii. Useful in high-risk populations iv. Complementary to PSA testing	i. Operator-dependent with poor inter-rater reliability ii. Limited reproducibility iii. Low sensitivity for early-stage disease	69–89%	84–98%	Minimally invasive	Chung & Hong (2025)
PSA	Measurement of serum PSA produced by prostate epithelial cells	i. Simple blood-based screening test ii. Inexpensive and widely available iii. Superior to DRE and TRUS for early detection	i. Low specificity ii. May lead to unnecessary biopsies iii. Elevated in benign conditions (*e.g.*, BPH, prostatitis) iv. No single optimal cutoff with high sensitivity and specificity	93%	20%	Non-invasive	Merriel et al. (2022)
PSAD	Ratio of serum PSA concentration to prostate volume	i. Simple and inexpensive ii. Improved specificity compared with PSA alone iii. Useful in the PSA “grey zone”	i. No consensus on optimal cutoff to avoid biopsy	74% (for 0.15 ng/mL cutoff)	61% (for 0.15 ng/mL cutoff)	Non-invasive	Wang et al. (2024)
Multiparametric MRI	Combination of T1-weighted, T2-weighted, diffusion-weighted, and dynamic contrast–enhanced imaging	i. Improved detection, localization, and staging of focal PCa	i. Reduced sensitivity for small or low-grade tumours ii. High cost and limited access in some settings iii. Require technical expertise and trained radiologists iv. May require contrast agents v. Inter-reader variability	93%	41%	Non-invasive	Ahmed et al. (2017)
TRUS/TP- biopsy	Ultrasound-guided systematic or MRI-targeted sampling of prostate tissue	i. Histopathological confirmation (reference standard) ii. Widely available and cost-effective	i. Sampling error may miss small or low-grade tumors ii. Risk of infection, bleeding, and discomfort	48%	96%	Invasive	Ahmed et al. (2017)
PSMA-PET	PET imaging using PSMA ligands (*e.g.*, ^68^Ga, ^18^F) targeting PSMA-expressing PCa cells	i. Superior detection of metastatic disease ii. High sensitivity even at low PSA levels (<0.5 ng/mL)	i. High cost ii. Limited availability	85%	98%	Non-invasive	Tsechelidis & Vrachimis (2022)
EPS-based biomarkers (proposed)	Analysis of expressed prostatic secretions collected after prostate massage (EPS-urine or direct EPS)	i. Prostate-proximal fluid enriched in prostate-derived proteins and EVs ii. Minimally invasive and repeatable iii. Potential for improved risk stratification *via* molecular profiling (cc, glycosylation patterns)	i. Pre-analytical variability (massage protocol, urine dilution) ii. Lack of standardized collection and analytical workflows iii. Clinical utility not yet prospectively validated	Not established	Not established	Minimally invasive	This review (Chew et al.)

**Notes.**

BPH benign prostatic hyperplasia DRE digital rectal examination EPS expressed prostatic secretion EV extracellular vesicle MRI magnetic resonance imaging PSA prostate-specific antigen PSAD prostate-specific antigen density PSMA-PET prostate-specific membrane antigen-positron emission tomography TP transperineal TRUS transrectal ultrasound

Across the disease course, better biomarkers are needed to refine risk stratification and monitor treatment response. Abiraterone with prednisolone, combined with androgen deprivation therapy (ADT), should be considered a new standard treatment for patients with high-risk non-metastatic PCa. In a metastatic setting, enzalutamide and abiraterone should not be combined for those starting long-term ADT. Clinically important improvements in survival from the addition of abiraterone to ADT are maintained for longer than 7 years, underlining the importance of accurate risk assessment and timely treatment adaptation (Attard et al., 2023). This review targets clinical chemists, urologists, cancer biologists, and biomarker and liquid biopsy researchers, and highlights EPS as a prostate-specific fluid biopsy matrix with potential for PCa diagnosis and longitudinal monitoring.

We focus on glycosylation alterations in EPS-urine, direct EPS, and EPS-derived extracellular vesicles (EVs), including global released-glycan profiling and glycoprotein-centric analyses such as PSA and prostatic acid phosphatase (PAP). We summarize current analytical strategies for EPS glycomics, link glycan features to disease state and aggressiveness, and discuss key technical and translational barriers, including pre-analytical variability and urine-related confounders. Finally, we outline opportunities to integrate EPS glycomics with other omics platforms for biomarker development and to contextualize EPS-derived signatures relative to serum, tissue, and cell-line glycomics.

## Survey Methodology

A literature search was performed in PubMed and Google Scholar to identify relevant publications up to June 2025. The strategy combined the keywords “expressed prostatic secretion”, “EPS”, “prostatic fluid”, “prostate cancer”, and “glycosylation” using appropriate Boolean operators. Original research articles, reviews, and clinical studies published between 1985 and 2025 in English were included. Studies were selected if they examined glycosylation differences between non-cancerous and PCa groups in EPS samples. Non-peer-reviewed articles, conference abstracts, and editorials were excluded to ensure scientific rigor and data reliability. Data extraction captured study objectives, analytical methodologies, principal findings, and conclusions, with particular emphasis on analytical quality and clinical relevance, to provide an up-to-date, overview of EPS glycosylation alterations in PCa.

### Liquid biopsies for cancer screening, diagnosis and monitoring

Liquid biopsy enables cancer detection and molecular characterization from body fluids, such as blood or urine, offering a minimally invasive alternative to tissue biopsy and allowing repeated sampling for longitudinal monitoring of tumor evolution and treatment response. In PCa, liquid biopsies, including serum- and urine-based assays, are particularly attractive due to their accessibility and potential to complement PSA testing by capturing multiple tumor-derived analytes, including circulating tumor cells, cell-free DNA, and EVs such as exosomes (Slomka et al., 2022). Beyond urine, serum biomarker development now extends past total PSA to incorporating androgen receptor variants such as AR-V7, markers of bone turnover, and neuroendocrine differentiation, as well as metabolomic signatures that may refine personalized risk assessment and treatment selection (Hamid et al., 2025). Exosomes are nanoscale, membrane-bound vesicles released by most cells that carry proteins, nucleic acids, and other cargo reflecting the molecular state of the originating tumor (Kalluri & LeBleu, 2020).

Urine-based liquid biopsy is emerging as a promising modality for PCa detection and risk stratification due to its non-invasive collection and anatomical proximity to the prostate. Systematic reviews highlight the diagnostic potential of urinary exosomal cargo, including microRNAs, proteins, and lipids, with reported utility for distinguishing PCa from benign prostatic hyperplasia (BPH) and for stratifying patients by disease aggressiveness (Hamid et al., 2025). More broadly, exosome analysis from blood, urine, and prostate-derived secretions provides opportunities for biomarker discovery and disease stratification while reducing dependence on invasive procedures (Khoo et al., 2021). In practice, however, serum PSA remains central for initial risk assessment but is limited by imperfect specificity, whereas urine assays are affected by dilution and non-prostatic background. Organ-proximal fluids such as EPS offer an intermediate matrix between serum and urine, enriched in prostate-derived proteins and EVs, yet still obtainable *via* post-DRE urine (EPS-urine) in outpatient settings. EPS-based biomarkers could refine biopsy decisions in men with elevated PSA and/or equivocal MRI, improve discrimination between indolent and aggressive disease, and, because EPS can be sampled repeatedly, support longitudinal monitoring during active surveillance by detecting molecular progression before overt clinical change.

### EPS and exosomes composition

In PCa, organ-proximal fluids such as EPS and seminal fluid are of particular interest because they are in direct contact with the prostate gland and may therefore reflect prostate physiology and pathology more than distal biofluids such as blood and urine (Drago et al., 2021). The prostate secretes prostatic fluid, which mixes with seminal vesicle secretions to support sperm activation and viability. EPS collected during or immediately after DRE contains prostate-derived proteins and EVs, making it a rich source of biomarkers (Drake et al., 2010). Two main EPS forms are used, including EPS-urine, obtained from first-void urine after prostate massage and direct EPS, collected intraoperatively from the urethral meatus (Graham et al., 2011).

Despite its relevance, EPS is relatively under-characterized due to limited sample availability, low biomolecule concentration and lack of standardized collection and analytical methods (Velonas et al., 2013). Metabolomic and proteomic studies of EPS-enriched urine consistently identify PSA and prostatic acid phosphatase (PAP) as abundant proteins with reduced levels in PCa compared with non-cancerous samples, while prostatic fluid is also notable for very high citrate concentration, reflecting specialized epithelial metabolism (Drake et al., 2009; Buszewska-Forajta et al., 2022). EPS-derived metabolomic signatures can discriminate PCa from BPH, although findings across matrices such as seminal fluid have been less consistent, underscoring the importance of sample type and biological context (Serkova et al., 2008; Roberts et al., 2017; Drago et al., 2021).

Beyond soluble proteins and metabolites, EPS contains abundant EVs, including exosomes, which are increasingly recognized as biomarker carriers. Exosomes are 30–100 nm vesicles that carry lipids, proteins, and RNA and contribute to angiogenesis, invasion and metastasis, immune evasion, and proliferation signaling in cancer (Kalluri & LeBleu, 2020). Prostasomes, a prostate-related EV subtype secreted by acinar epithelial cells, are abundant in prostatic secretion, seminal fluid, and ejaculate. In addition to their reproductive functions, they have been implicated in promoting tumor cell proliferation and invasion in PCa models (Ramirez-Garrastacho et al., 2022; Urabe et al., 2024). Their high physicochemical stability, protection of cargo from enzymatic degradation, and selective molecular packaging make them attractive candidates for diagnostic and prognostic biomarker discovery (Zijlstra & Stoorvogel, 2016; Larssen et al., 2017; Dang et al., 2020).

EV preparations are, however, heterogeneous and comprise multiple subpopulations that differ in composition, biogenesis, and function, including in direct EPS and EPS-urine (Nolan & Duggan, 2018). This is largely because commonly used isolation methods do not readily distinguish EV subtypes, and most preclinical studies therefore analyze pooled vesicle populations, potentially obscuring the features of rare or functionally distinct subsets (Shao et al., 2018). This issue is compounded in urine-based EV isolation by variability in pH, ionic strength, and protein composition, as well as interference from uromodulin (Tamm-Horsfall protein, THP), which can entrap EVs and reduce recovery or co-isolate with vesicles and compromise downstream proteomics and glycomics analyses (Xu et al., 2019; Micanovic et al., 2020). Optimized workflows incorporating dithiothreitol and alkaline washing during differential ultracentrifugation can disrupt the THP networks, improve EV purity and increase recovery from EPS-urine, thereby enhancing detection of low-abundance vesicular glycoproteins in prostate-derived samples (Correll et al., 2022).

### EPS: collection and processing

EPS can be clinically collected after DRE-induced prostate massage and represents a valuable biofluid for PCa diagnosis and monitoring because it more directly reflects prostate-specific pathological and functional changes than blood or urine (Blaschke et al., 2021). During prostate massage, prostatic fluids and detached epithelial cells are displaced into the urethra and collected in the first voided urine, which is predominantly prostate-derived with minimal seminal vesicle or sperm contamination. DRE does not substantially alter serum PSA, whereas urinary PSA increases significantly post-DRE, supporting the use of EPS-urine as an organ-proximal matrix (Shrivastava et al., 2020).

The measured EPS glycome may be influenced by collection-related and other pre-analytical factors, particularly whether analytes are obtained as direct EPS, EPS-urine, or an EV-enriched fraction. Direct comparative profiling has shown that direct EPS contains higher levels of sialylated non-fucosylated *N-*glycans, whereas EPS-urine is enriched in fucosylated and tetra-antennary species (Blaschke et al., 2021). More broadly, urinary EV guidance suggests that DRE conditions and urine fraction can affect vesicle yield and apparent glycoprofiles (Erdbrugger et al., 2021). In PCa, post-DRE urine biomarker yields may also vary according to the DRE technique used (Smith et al., 2024). Similarly, Nyalwidhe et al. (2013) showed that exosome-derived EPS profiles were broadly similar to whole-fluid EPS but relatively enriched in large tetra-antennary glycans, suggesting that the fractionation strategy can influence the apparent representation of glycan classes. Taken together, the available literature suggests that EPS collection format (direct EPS *versus* EPS-urine) and DRE-associated sampling conditions may influence the apparent glycome. However, direct evidence that a specific EPS collection maneuver consistently causes selective loss or underrepresentation of a defined glycan structure remains limited.

After collection, EPS-urine (typically 10–20 mL) is kept on ice, cleared by centrifugation to remove debris, and the clarified supernatant is aliquoted, and stored at −80 °C, whereas direct EPS yields smaller volumes (about 0.5–1 mL) expressed from the urethral meatus (Nyalwidhe et al., 2013). EV enrichment from EPS follows workflows used for other biofluids. Differential ultracentrifugation remains the most common approach, but it requires specialized high-speed equipment (ultracentrifuge capable of ∼100,000 to 200,000×*g*), being suited to relatively small volumes (Coughlan et al., 2020). Density-gradient centrifugation, using media such as iodixanol, can further improve purity by separating EVs by buoyant density, but adds time, reduces yield, and is less practical for routine clinical use (Brennan et al., 2020).

Ultrafiltration (usually 100–300 kDa cut-off) and polymer-based precipitation offer faster, more scalable alternatives for high-throughput or exploratory workflows, but polyethylene glycol-based precipitation can cause vesicle aggregation and co-precipitation of lipoproteins and other non-vesicular components that complicate downstream proteomics and glycomics (Ansari et al., 2024). Overall, no single isolation strategy is universally optimal. Rapid precipitation methods may be appropriate for screening or clinical assays, whereas omics applications that are highly sensitive to contaminants, including glycomics, generally require higher-purity preparations based on ultracentrifugation, or can achieve even greater purity when ultracentrifugation is combined with the density gradient method (Brennan et al., 2020; Coughlan et al., 2020).

EV isolation methodology can influence downstream glycomics data. In a direct comparative study, ultracentrifugation, density-gradient centrifugation, and size-exclusion chromatography yielded broadly similar EV glycoprofiles, whereas polymer-based precipitation produced the most distinct profile (Freitas et al., 2019). Consistent with this, urinary EV guidance notes that isolation workflows affect EV yield, purity, and co-isolated contaminants, which may in turn influence downstream molecular readouts (Erdbrugger et al., 2021). Glycan-targeted affinity approaches are also inherently selective. For example, lectin-based capture can enrich high-mannose EVs and thereby skew the apparent representation of glycan classes (Yamamoto et al., 2019). However, direct evidence remains limited as to whether any routine isolation method consistently biases detection against specific glycan structures across EV sources, and this should therefore be recognized as a current methodological limitation.

### Glycosylation

Glycosylation is an essential post-translational modification that generates a diverse, tightly regulated repertoire of cellular glycans involved in adhesion, trafficking, receptor activation, signal transduction, endocytosis and molecular clearance in health and disease (He, Zhou & Wang, 2024). The two principal classes of protein glycosylation are *N-* and *O-* glycosylation, which are coordinated by enzyme networks in the endoplasmic reticulum (ER) and Golgi apparatus and strongly influence glycoprotein structure, stability, and function (Wang et al., 2019). *N-*glycans share a conserved Man_3_GlcNAc_2_ core and are categorized as oligomannose, hybrid, or complex types (Kizuka & Taniguchi, 2016). They are built from a dolichol-linked precursor in the ER, then trimmed and remodeled in the Golgi (Schoberer et al., 2018). In contrast, *O-*glycans are structurally more heterogenous, with mucin-type *N-*acetylgalactosamine (GalNAc) *O-*glycosylation predominating in mammals and being initiated in the Golgi by GalNAc-transferases (GnT) (Zhang et al., 2022). Mucin-type *O-*GalNAc glycans are classified into eight core structures (cores 1–8), of which cores 1–4 are the most common. Biosynthesis is initiated by the addition of GalNAc*α*1-*O-*Ser/Thr, known as the Tn antigen. This precursor may be extended to form core 1 structure (Gal*β*1-3GalNAc*α*1-Ser/Thr) by C1GALT1 or core 3 (GlcNAc*β*1-3GalNAc*α*1-Ser/Thr) by *β*3GnT6. Further modifications of core 1 and core 3 give rise to core 2 (GlcNAc*β*1-6(Gal*β*1-3)GalNAc*α*1-Ser/Thr) and core 4 (GlcNAc*β*1-6(GlcNAc*β*1-3)GalNAc*α*1-Ser/Thr), respectively, whereas the glycosyltransferases responsible for the synthesis of cores 5–8 remain incompletely characterized (Zhang et al., 2022). Typical *N-* and *O-*glycans contain about 10–12 residues drawn from galactose, GlcNAc, GalNAc, mannose, fucose, and occasionally xylose, and may include *N-*acetylneuraminic acid (Neu5Ac) and *N-*glycolylneuraminic acid (Neu5Gc), depending on species and context (Xu et al., 2024).

**Figure 1:**
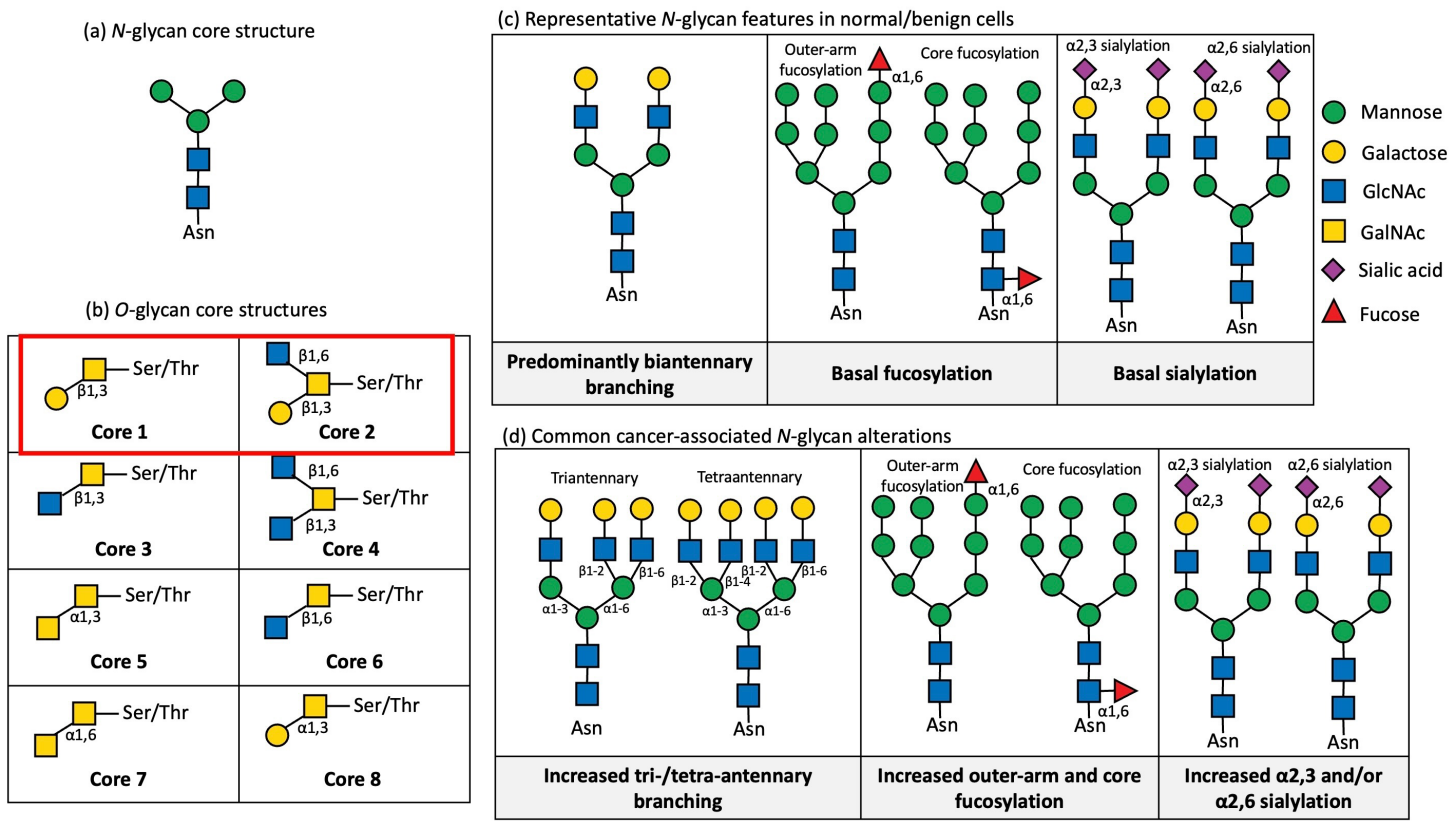
*N-* and *O-*glycan core structures and representative glycosylation changes associated with cancer. (A) Conserved *N-*glycan core structure linked to Asn. (B) The eight major *O-*glycan core structures linked to Ser/Thr, with Core 1 and Core 2 highlighted as the *O*-glycan cores most frequently associated with tumor-related glycosylation changes discussed in this review. (C) Representative *N*-glycan features in normal/benign cells are characterized predominantly by biantennary branching with physiological levels of fucosylation and sialylation. (D) Common cancer-associated *N-*glycan alterations, including increased tri- and tetra-antennary branching, increased outer-arm and core fucosylation, and increased *α*2,3- and/or *α*2,6-linked sialylation. Glycosidic linkages are indicated for the illustrated aberrant structures, and the schematics represent relative glycosylation changes associated with malignancy rather than cancer-specific structures.

Glycans are conventionally represented using Symbol Nomenclature for Glycans (SNFG), which not only aids intuitive visualization but also facilitates algorithm development and standardized communication across glycoinformatics platforms (Neelamegham et al., 2019). Figure 1 summarizes the conserved N-glycan core, the eight mucin-type *O-*glycan core structures, and selected cancer-associated glycan alterations relevant to this review, adapted with reference to established glycan nomenclature and structural features (Varki et al., 2022). In particular, core 1 and core 2 are highlighted as the *O-*glycan cores most commonly associated with tumor cells in the studies discussed in this review.

In biological systems, glycosylation contributes to protein folding, ER quality control, stability, trafficking, and localization, and altered *N-*glycosylation can impair membrane targeting and protein function (Jayaprakash & Surolia, 2017). At the cell surface, glycosylation of adhesion molecules and receptors regulates adhesion complex stability and downstream signaling, while glycan–lectin interactions involving galectins, Siglecs, and C-type lectins can promote either immune activation or immune suppression (Smith & Bertozzi, 2021).

In cancer, glycosylation is frequently remodeled by both intrinsic oncogenic programs and extrinsic tumor microenvironmental cues. Hypoxia and inflammation can alter glycosyltransferase activity and metabolic flux through pathways and metabolism such as the hexosamine biosynthesis pathway (HBP), vascular endothelial growth factor and intercellular adhesion molecule 1, thereby altering glycan changes such as reduced *α*2,6-sialylation, increased *β*1,6-GlcNAc branching, and accumulation of poly-*N-*acetyllactosamine to support angiogenesis, tumor cell interactions and immune evasion (Lucena et al., 2016; Laubli & Borsig, 2019; Pietrobono & Stecca, 2021).

Aberrant glycosylation is a recognized hallmark of malignancy and refers to disease-associated changes in glycan structures, including altered sialylation, fucosylation, and branching (He, Zhou & Wang, 2024). These changes can generate tumor-associated carbohydrate antigens (TACAs), which are cancer-associated glycan epitopes expressed on tumor cell surfaces and linked to tumor progression, immune modulation, and biomarker potential. Cancer-associated glycans may arise through incomplete biosynthesis, producing truncated mucin-type *O-*glycans such as Tn, T, sialyl-Tn, and sialyl-T, or through neo-synthesis of structures such as Lewis antigens and their sialylated derivatives (sLe_a_ and sLe_x_), which influence adhesion, invasion and immune recognition (Van de Wall et al., 2020; Pinto & Parameswaran, 2023).

These concepts are directly relevant to PCa. Sialylated glycans are frequently enriched in PCa (Scott et al., 2023), making increased sialylation a representative example of aberrant glycosylation and the resulting sialylated glycan epitopes potential TACAs in this disease context. In parallel, dysregulated expression of glycosyltransferases, including *β*-galactosidase-*α*2,6-*N-* sialyltransferase 1 (ST6Gal1), *β*-galactosidase-*α*2,3-*N*-sialyltransferase 1 (ST3Gal-1) and fucosyltransferase 8 (FUT8), has been associated with increased *α*2,6- and *α*2,3-sialylation and increased core fucosylation, which downstream effects on receptor signaling, epithelial-mesenchymal transition (EMT), and immune escape (Bastian et al., 2021; Garnham et al., 2024). Enhanced *β*1,6-branching driven by MGAT5 promotes tri- and tetra-antennary structures that influence receptor retention and signaling strength, whereas MGAT3-mediated bisecting GlcNAc may stabilize adhesion and reduce metastatic potential (Taniguchi et al., 2021). Glycan biosynthesis reflects the combined activity of multiple enzymes and sensitively captures disease-associated changes that are not always evident at the gene or total protein level, making glycomics a particularly informative layer for biomarker discovery in PCa (Mereiter et al., 2019).

### Strategies for glycomic profiling

Glycan profiling can be tailored from intact glycoprotein analysis to global characterization of released glycans from complex matrices (Mereiter et al., 2019). Bottom-up glycoproteomics examines glycopeptides generated by proteolysis, enabling protein identification and site-specific microheterogeneity. Microheterogeneity is where a single glycosylation site may be occupied by multiple distinct glycan structures, resulting in heterogenous populations of glycoforms rather than a single uniform modification. Alternatively, released-glycan analysis, either enzymatically or chemically, liberates glycans from proteins to survey the overall glycome (Delafield & Li, 2021). Peptide-*N-*glycosidase F is widely used for most *N-*glycans, whereas peptide-*N*-glycosidase A is needed for core *α*1,3-fucosylated structures. *O-*glycans usually require chemical release because no single enzyme addresses their full diversity (Wilkinson & Saldova, 2020). However, *O-*glycosidase specifically cleaves unsubstituted core 1 *O*-glycan structures (Gal*β*1-3GalNAc*α*Ser/Thr) and does not efficiently release more extended or substituted *O-*glycans such as core 2 structures. To address this limitation, exoglycosidases may be used sequentially to trim terminal residues and simplify glycan structures before analysis. Although this strategy can facilitate *O-*glycan profiling while preserving protein integrity, detailed structural elucidation of intact and complex *O-*glycans remains analytically challenging.

Glycoprofiling typically combines separation and detection to generate characteristic glycan signatures. High-performance or ultra-performance liquid chromatography (HPLC/UPLC) of fluorescently labelled glycans, often using HILIC, remains central for quantitative and comparative work, with alternative modes such as weak anion exchange and reversed-phase adding charge- or hydrophobicity-based resolution (Messina et al., 2021). Capillary electrophoresis with laser-induced fluorescence (CE-LIF) offers high efficiency and, in multicapillary formats, higher throughput for charge-to-size-based separation of *N-*glycans (Li et al., 2022). Mass spectrometry (MS) underpins modern glycomics by providing accurate masses and, *via* tandem MS, could provide structural information (De Haan et al., 2022). Matrix-assisted laser desorption/ionization time-of-flight (MALDI-ToF) is widely used for rapid fingerprinting (Kumar, 2024), whereas LC-electrospray ionization (ESI)-MS/MS with tailored fragmentation is required for confident linkage and stereochemical assignments for glycopeptide-level analysis (Molnarova et al., 2022).

In bottom-up glycoproteomics, LC-ESI-MS/MS of glycopeptides uses fragmentation methods such as HCD, ETD and EThcD to balance information of glycan and peptide backbones, with HCD favoring glycosidic cleavages and diagnostic oxonium ions, and ETD/ EThcD providing improved peptide sequencing and site localization (Riley et al., 2020). Data analysis remains more labor-intensive than in standard proteomics, but tools such as GlycoMod and GlycoWorkbench and curated resources like UniCarb-DB and UniCarb-KB support composition prediction, spectral annotation and dataset sharing (Cooper, Gasteiger & Packer, 2001; Ceroni et al., 2008; Campbell et al., 2014). Lectin microarrays offer a rapid, high-throughput means of motif-level profiling directly on intact glycoproteins and are particularly valuable for low-abundance or highly diluted samples. Lectin-based methods lack the structural resolution of MS and is best used in combination with MS-based workflow (Yu et al., 2020).

**Figure 2:**
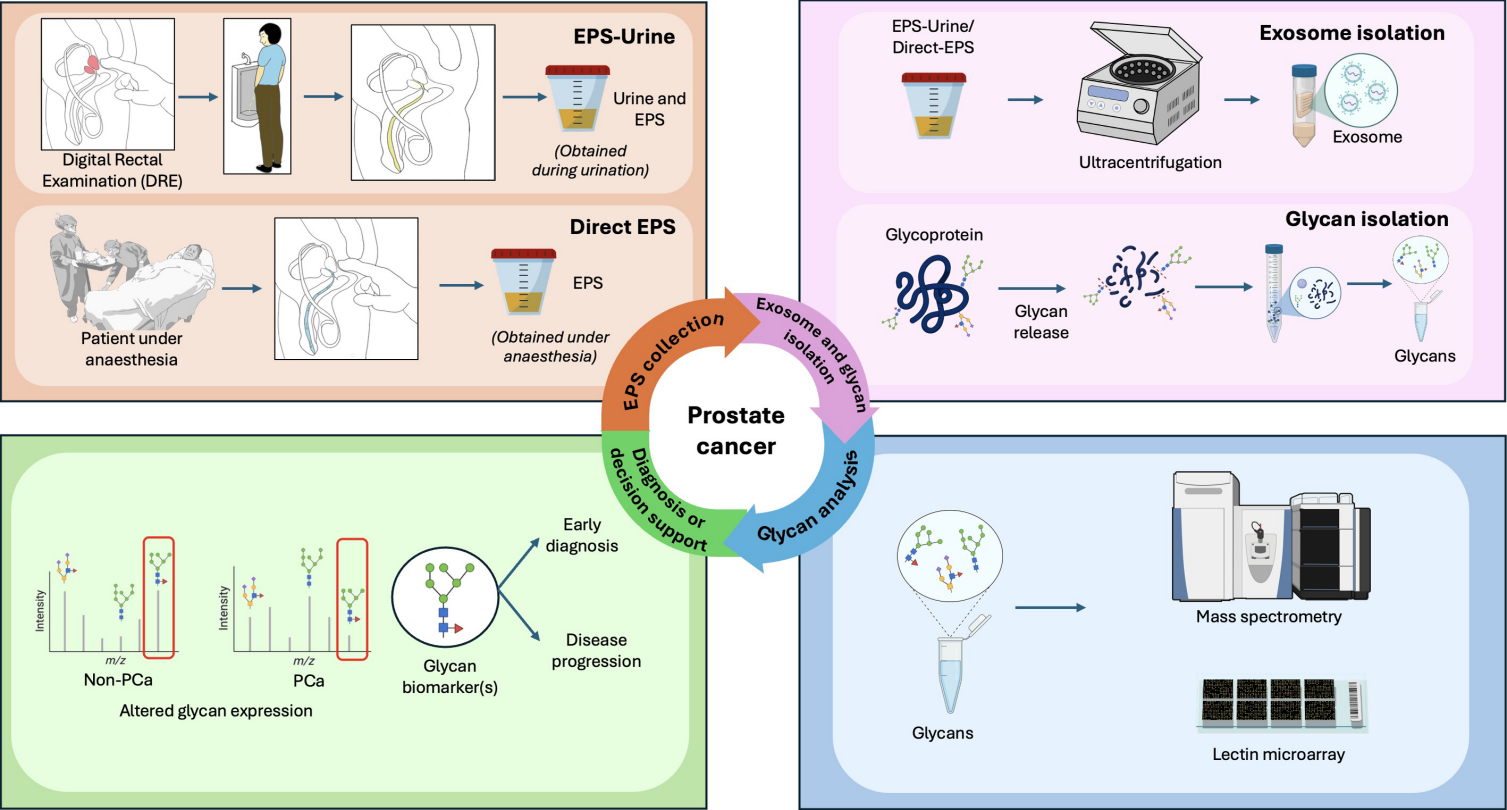
Integrated workflow for EPS-based glycomics in PCa. Schematic overview of EPS collection (EPS-urine after prostate massage and/or direct EPS), EV (exosome/small EV) enrichment, glycoprotein/glycan preparation (released-glycan or glycoprotein-centric workflows), and downstream glycan/glycopeptide profiling to support PCa biomarker discovery.

EPS is a highly complex and dilute matrix because prostatic fluids are further diluted upon mixing with urine. In this context, lectin-based enrichment strategies are especially useful, as they provide motif-level information and selectively increase the relative abundance of fucosylated and sialylated glycoproteins before downstream separation and MS analysis. Combining EPS-specific enrichment with sensitive LC-MS/MS or CE-LIF platforms can therefore enhance detection of prostate-derived glycan alterations against the urinary background and is well-suited to discovering clinically relevant glycomic biomarkers. Figure 2 schematically summarizes the EPS-to-glycomics workflow, from collection and EV enrichment through glycan release or enrichment to analytical profiling.

Glycomics data are typically quantified using integrated chromatographic peak areas or MS ion intensities and expressed as relative abundances within each sample. Although total-area normalization is widely used, glycomics data are compositional in nature, and log-ratio transformations such as centered log-ratio or additive log-ratio are increasingly recommended for group comparisons (Bennett et al., 2025). When more robust quantification is required, stable-isotope-labeled or internal glycan standards may be used (Mechref et al., 2013). Disease-associated changes are then identified by comparing individual glycans or derived traits, such as total fucosylation, sialylation, branching, or bisection, between groups using appropriate statistical tests, such as t-tests or Mann–Whitney U tests for two-group comparisons, with multiple-testing correction applied where necessary. For diagnostic panel development, multivariable or logistic regression and receiver operating characteristic or area under the curve are commonly used, with validation (Diaz-Fernandez et al., 2024). If only one or two glycomics-related parameters are measured, such as total fucose, they are analyzed similarly as continuous variables after appropriate normalization. Universal normal reference values are generally unavailable because glycomics traits vary with age, sex, ethnicity, specimen type, and analytical platform. Most studies, therefore, interpret abnormalities relative to matched controls or cohort-specific reference distributions (Ding et al., 2011; Gebrehiwot et al., 2018).

### Glycosylation alterations in EPS and EPS-derived EVs in PCa

Drake et al. (2009) first profiled PSA and PAP glycoproteins in EPS, identifying prostate-derived glycoforms with potential diagnostic utility and establishing EPS as a rich source of prostate-associated glycoproteins. Subsequent work has extended PSA glycan characterization to serum, PCa cell lines, and seminal fluids, but direct EPS and EPS-urine remain particularly attractive because PSA concentrations are markedly higher than in serum, enabling detailed glycan analysis that is more relevant to early detection (Tajiri, Ohyama & Wada, 2008; Jia et al., 2017).

Global EPS-urine glycoprofiling and PSA-focused glycomics have revealed complementary but not identical cancer-associated patterns. Table 2 summarizes reported EPS-associated glycosylation alterations across cohorts, platforms, and clinical groupings. Vermassen et al. (2015) reported reduced core fucosylation and lower triantennary *N-* glycans in EPS-derived urinary glycoproteins from PCa compared with benign and healthy groups, with further decreases in high-risk disease, suggesting that loss of core fucosylation may mark progression. In contrast, Jia et al. (2017) purified PSA from EPS-urine and observed increased core-fucosylated, diasialylated biantennary glycans in PCa *versus* BPH, and progressive shifts in PSA glycoforms with increasing Gleason score, indicating that protein-specific changes can diverge from global trends yet still provide strong diagnostic and prognostic signals (Gratacos-Mulleras et al., 2020).

**Table 2:** Summary of glycosylation studies using EPS to distinguish benign from prostate cancer (PCa) cases and to assess disease progression. The table highlights consistent alterations in *N-*glycan branching and core fucosylation across studies, as well as compartment-specific differences between bulk EPS and EPS-derived EVs.

**Study**	**Study groups**	**Sample size**	**Detection method**	**Samples**	**Clinical endpoint**	**Key glycosylation alterations**	**Representative glycan structures/motifs**	
**Clinical utility: Diagnosis**								
Vermassen et al. (2015) ^*^	Healthy, BPH, PCa	Healthy, *n* = 54; BPH, *n* = 93; PCa, *n* = 74	Multicapillary electrophoresis (ABI3130; UGM score)	EPS-urine (Total *N*-glycome)	PCa * vs* benign (healthy + BPH)	i. ↓ Core fucosylation of multiantennary *N*-glycan	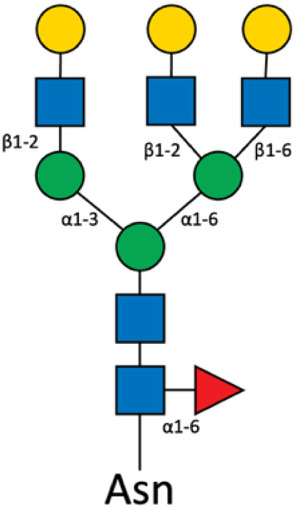 Gal*β*1-4GlcNAc*β*1-2Man*α*1-3[Gal*β*1-4GlcNAc*β*1-2(Gal*β*1-4GlcNAc*β*1-6)Man*α*1-6]Man*β*1-4GlcNAc*β*1-4(Fuc*α*1-6)GlcNAc-Asn	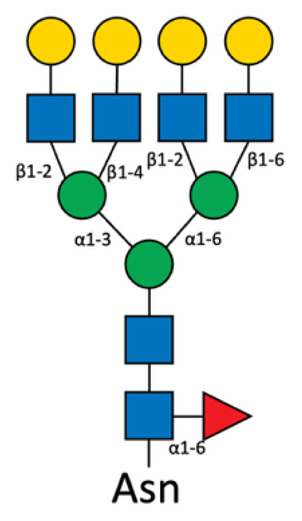 Gal*β*1-4GlcNAc*β*1-2Man*α*1-3(Gal*β*1-4GlcNAc*β*1-4)[Gal*β*1-4GlcNAc*β*1-2(Gal*β*1-4GlcNAc*β*1-6)Man*α*1-6]Man*β*1-4GlcNAc*β*1-4(Fuc*α*1-6)GlcNAc-Asn
						iii. ↓ Total abundance of triantennary *N*-glycans	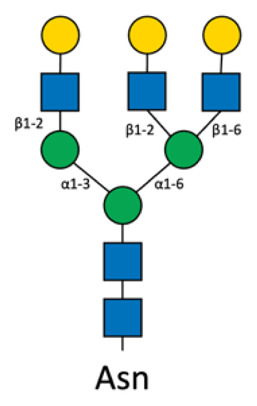 Gal*β*1-4GlcNAc*β*1-2Man*α*1- 3[Gal*β*1-4GlcNAc*β*1-2 (Gal*β*1-4GlcNAc*β*1 -6)Man*α*1-6]Man*β*1 -4GlcNAc*β*1-4GlcNAc-Asn	
Jia et al. (2017)	BPH * vs* PCa	BPH *n* = 32; PCa *n* = 30	UPLC-FLD/ QTOF-MS	EPS-urine (PSA-focused glycomics)	PCa * vs* BPH	i. ↑ Core fucosylated PSA glycans	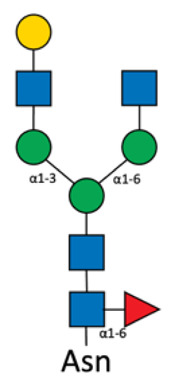 Gal*β*1-4GlcNAc*β*1-2Man*α*1-3[GlcNAc*β*1-2Man*α*1-6]Man*β*1-4GlcNAc*β*1-4(Fuc*α*1-6)GlcNAc-Asn	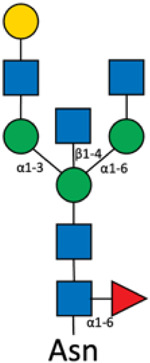 Gal*β*1-4GlcNAc*β*1-2Man*α*1-3[GlcNAc*β*1-2Man*α*1-6][GlcNAc*β*1-4]Man*β*1-4GlcNAc*β*1-4(Fuc*α*1-6)GlcNAc-Asn
						ii. ↑ Core fucosylated, monosialylated, biantennary hybrid PSA glycans	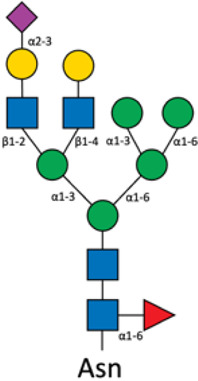 NeuAc*α*2-3Gal*β* 1-4GlcNAc*β*1- 2Man*α*1-3[Man*α*1 -3(Man*α*1-6)Man*α*1 -6][GlcNAc*β*1-4]Man*β*1 -4GlcNAc*β*1 -4(Fuc*α*1-6)GlcNAc-Asn	
**Clinical utility: Progression**								
Vermassen et al. (2015)	Gleason scores <7, = 7, >7	GS 6, *n* = 19; GS 7 *n* = 36; GS ≥ 8 *n* = 19	Multicapillary electrophoresis (ABI3130; UGM score)	EPS-urine (Total *N*-glycome)	Risk stratification by GS	i. ↓ Core fucosylation of multiantennary *N*-glycans	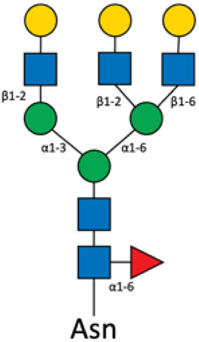 Gal*β*1-4GlcNAc*β*1-2Man*α*1-3[Gal*β*1-4GlcNAc*β*1-2(Gal*β*1-4GlcNAc*β*1-6)Man*α*1-6]Man*β*1-4GlcNAc*β*1-4(Fuc*α*1-6)GlcNAc-Asn	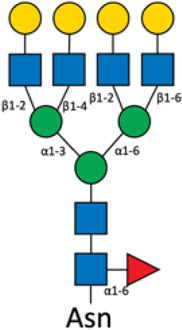 Gal*β*1-4GlcNAc*β*1-2Man*α*1-3[Gal*β*1-4GlcNAc*β*1-4][Gal*β*1-4GlcNAc*β*1-2(Gal*β*1-4GlcNAc*β*1-6)Man*α*1-6]Man*β*1-4GlcNAc*β*1-4(Fuc*α*1-6)GlcNAc-Asn
						ii. ↓ Core fucosylation of biantennary *N*-glycan	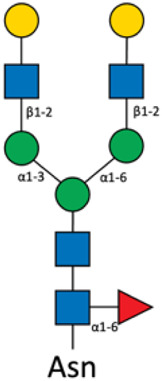 Gal*β*1-4GlcNAc*β*1 -2Man*α*1-3[Gal*β*1 -4GlcNAc*β*1-2Man*α*1-6]Man*β*1 -4GlcNAc*β*1-4(Fuc*α*1-6)GlcNAc-Asn	
Nyalwidhe et al. (2013)	Benign, low-risk, PCa, high-risk PCa	*n* = 10 per group	Lectin profiling; FT-ICR MALDI-TOF	EPS-urine (Total *N*-glycome)	Low- * vs* high-grade PCa	i. ↓ High-mass tri- and tetra-antennary with *N*-glycans	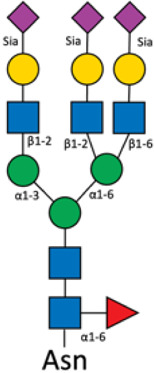 NeuAc?-Gal*β*1-4GlcNAc*β*1-2Man*α*1-3[NeuAc?-Gal*β*1-4GlcNAc*β*1-2(NeuAc?-Gal*β*1-4GlcNAc*β*1-6)Man*α*1-6]Man*β*1-4GlcNAc*β*1-4(Fuc*α*1-6)GlcNAc-Asn	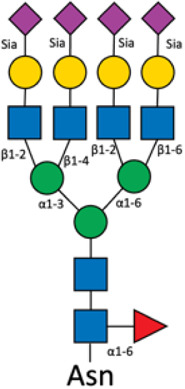 NeuAc?-Gal*β*1-4GlcNAc*β*1-2Man*α*1-3(NeuAc?-Gal*β*1-4GlcNAc*β*1-4)[NeuAc?-Gal*β*1-4GlcNAc*β*1-2(NeuAc?-Gal*β*1-4GlcNAc*β*1-6)Man*α*1-6]Man*β*1-4GlcNAc*β*1-4(Fuc*α*1-6)GlcNAc-Asn
						ii. ↓ Fucosylation and sialylation	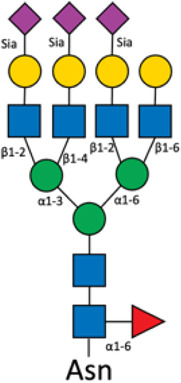 NeuAc?-Gal*β*1-4GlcNAc*β*1-2Man*α*1-3(NeuAc?-Gal*β*1-4GlcNAc*β*1-4)[NeuAc?-Gal*β*1-4GlcNAc*β*1-2(Gal*β*1-4GlcNAc*β*1-6)Man*α*1-6]Man*β*1-4GlcNAc*β*1-4(Fuc*α*1-6)GlcNAc-Asn	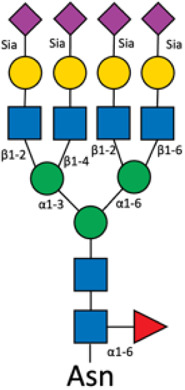 NeuAc?-Gal*β*1-4GlcNAc*β*1-2Man*α*1-3(NeuAc?-Gal*β*1-4GlcNAc*β*1-4)[NeuAc?-Gal*β*1-4GlcNAc*β*1-2(NeuAc?-Gal*β*1-4GlcNAc*β*1-6)Man*α*1-6]Man*β*1-4GlcNAc*β*1-4(Fuc*α*1-6)GlcNAc-Asn
				EPS-derived EV (Total *N*-glycome)	Low- * vs* high-grade PCa	i. ↑ Biantennary *N*-glycan with bisecting GlcNAc	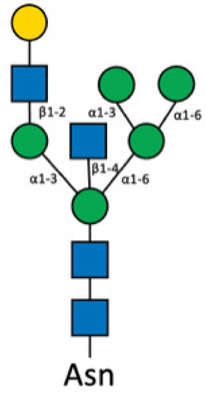 Gal*β*1-4GlcNAc*β*1-2Man*α*1- 3[Man*α*1-3(Man*α*1 -6)Man*α*1-6][GlcNAc*β*1- 4]Man*β*1- 4GlcNAc*β*1-4GlcNAc-Asn	
						ii. ↑ High-mass tri- and tetra-antennary with *N*-glycans	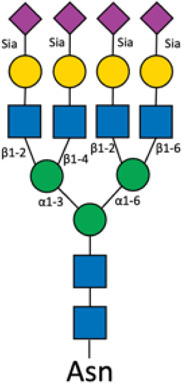 NeuAc?-Gal*β*1-4GlcNAc*β*1-2Man*α*1-3(NeuAc?-Gal*β*1-4GlcNAc*β*1-4)[NeuAc?-Gal*β*1-4GlcNAc*β*1-2(NeuAc?-Gal*β*1-4GlcNAc*β*1-6)Man*α*1-6]Man*β*1-4GlcNAc*β*1-4GlcNAc-Asn	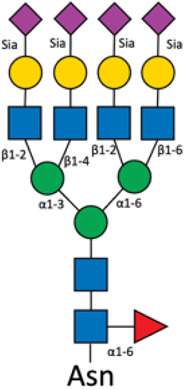 NeuAc?-Gal*β*1-4GlcNAc*β*1-2Man*α*1-3(NeuAc?-Gal*β*1-4GlcNAc*β*1-4)[NeuAc?-Gal*β*1-4GlcNAc*β*1-2(NeuAc?-Gal*β*1-4GlcNAc*β*1-6)Man*α*1-6]Man*β*1-4GlcNAc*β*1-4(Fuc*α*1-6)GlcNAc-Asn
				Direct EPS (Total *N*-glycome)	Low- * vs* high-grade PCa	i. ↓ High-mass tri- and tetra-antennary with *N*-glycans	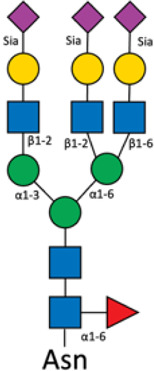 NeuAc?-Gal*β*1-4GlcNAc*β*1-2Man*α*1-3[NeuAc?-Gal*β*1-4GlcNAc*β*1-2(NeuAc?-Gal*β*1-4GlcNAc*β*1-6)Man*α*1-6]Man*β*1-4GlcNAc*β*1-4(Fuc*α*1-6)GlcNAc-Asn	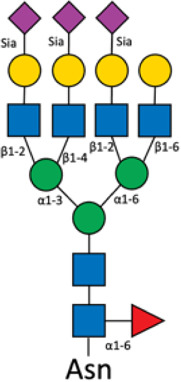 NeuAc?-Gal*β*1-4GlcNAc*β*1-2Man*α*1-3(NeuAc?-Gal*β*1-4GlcNAc*β*1-4)[NeuAc?-Gal*β*1-4GlcNAc*β*1-2(Gal*β*1-4GlcNAc*β*1-6)Man*α*1-6]Man*β*1-4GlcNAc*β*1-4(Fuc*α*1-6)GlcNAc-Asn
Jia et al. (2017) ^*^	Low * vs* high Gleason score	*n* = 15 per group	UPLC-FLD/ QTOF-MS	PSA-focused glycomics	Low- * vs* high-risk PCa	i. ↑ Core fucosylated biantennary PSA glycans	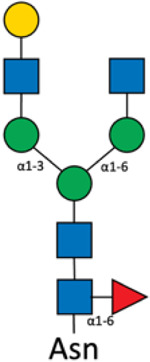 Gal*β*1-4GlcNAc*β*1-2Man*α*1-3[GlcNAc*β*1-2Man*α*1-6]Man*β*1-4GlcNAc*β*1-4(Fuc*α*1-6)GlcNAc-Asn	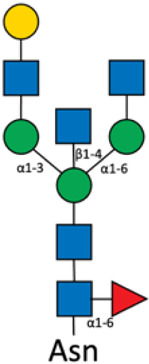 Gal*β*1-4GlcNAc*β*1-2Man*α*1-3[GlcNAc*β*1-2Man*α*1-6][GlcNAc*β*1-4]Man*β*1-4GlcNAc*β*1-4(Fuc*α*1-6)GlcNAc-Asn
						ii. ↓ Di-sialylated biantennary PSA glycans	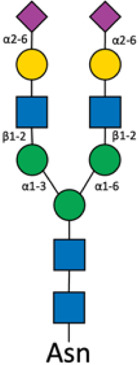 NeuAc*α*2-6Gal*β*1-4GlcNAc*β*1-2Man*α*1-3[NeuAc*α*2-6Gal*β*1-4GlcNAc*β*1-2Man*α*1-6]Man*β*1-4GlcNAc*β*1-4GlcNAc-Asn	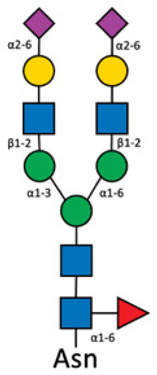 NeuAc*α*2-6Gal*β*1-4GlcNAc*β*1-2Man*α*1-3[NeuAc*α*2-6Gal*β*1-4GlcNAc*β*1-2Man*α*1-6]Man*β*1-4GlcNAc*β*1-4(Fuc*α*1-6)GlcNAc-Asn

**Notes.**

↑ indicate higher relative abundance in the PCa compared to the benign or lower-risk group.

↓ indicate lower relative abundance in the PCa compared to the benign or lower-risk group.

* indicates statistically significant differences (*p* ≤ 0.05).

BPH benign prostatic hyperplasia Direct EPS direct expressed prostatic secretion EPS-derived EV expressed prostatic secretion-derived extracellular vesicle EPS-urine expressed prostatic secretion-urine FT-ICR MALDI Fourier transform ion cyclotron resonance matrix-assisted laser desorption/ionization time-of-flight GS Gleason score MALDI-IMS-QTOF matrix-assisted laser desorption/ionization imaging mass spectrometry coupled with quadrupole time-of-flight UGM urinary glycoprofile marker UPLC-FLD/ QTOF-MS ultra-performance liquid chromatography coupled with fluorescence detection and quadrupole time-of-flight mass spectrometry

Nyalwidhe et al. (2013) extended these analyses to direct EPS and EPS-derived EVs and found that high-mass tri- and tetraantennary *N-*glycans and fucosylation and sialylation were decreased in EPS-urine and direct EPS from advanced disease, consistent with Vermassen et al. (2015), suggesting urine dilution may not obscure prostate-derived glycomics changes and supporting EPS-urine as a practical alternative to direct EPS. However, this is based on limited data and requires validation in larger cohorts. EPS-derived EVs from high-risk patients showed relative enrichment of high-mass tri- and tetra-antennary structures, implying that EVs form a distinct glycomics compartment. These opposing trends may arise from selective EV cargo packaging and enrichment of membrane glycoproteins (Royo et al., 2016), but may also reflect variability in EV isolation purity and co-isolated urinary glycoproteins such as uromodulin (Correll et al., 2022). Standardized isolation procedures and orthogonal EV characterization are therefore essential before these compartment-specific patterns can be interpreted biologically (De Sousa et al., 2023). Blaschke et al. (2021) further compared glycosylation across EPS-urine, direct EPS, and EPS-derived EVs, identifying distinct profiles for each matrix. Overall, most glycans were fucosylated and/or biantennary. Direct EPS and EPS-derived EVs showed similar glycan profiles, suggesting a large fraction of EPS glycoproteins may be vesicle-associated. This contrasts with Nyalwidhe et al. (2013), who found direct EPS more closely resembled EPS-urine than EPS-derived EV. However, Blaschke et al. (2021) did not clearly report donor clinical status or benign–malignant and grade-related differences, and their small cohort (*n*= 10 per group) limits interpretation of disease-associated patterns. They also observed more high-mannose structures in EPS-derived EV than in EPS-urine or direct EPS.

Jia et al. (2017) additionally examined EPS-associated glycosylation in relation to progression by focusing on PSA purified from EPS-urine. They reported reduced disialylated biantennary structure in high-grade disease, aligning with decreased sialylation in EPS-urine described by Nyalwidhe et al. (2013), and increased core-fucosylated biantennary glycans in high Gleason scores *versus* low scores, mirroring differences between PCa and benign controls. This pattern contrasts with the decreased core fucosylation reported during progression (Vermassen et al., 2015). These discrepancies likely reflect differences in analytical targets and biological context. PSA-centric analyses capture changes on a single abundant prostate-derived protein, whereas global EPS profiling averages signals across many glycoproteins with distinct expression patterns and glycosylation machinery. As glycosylation is non-templated and highly responsive to microenvironmental cues (Pasala et al., 2024), both strategies can yield informative but non-identical views of disease state. Global EPS profiling offers a broad survey of disease-associated glycan alterations, while PSA-focused glycomics may provide a more targeted and interpretable biomarker panel; together, they are complementary in biomarker discovery.

Across available studies, EPS-urine, direct EPS, and EPS-derived EV all show promise as PCa-relevant liquid biopsy sources, but direct comparison is hindered by methodological heterogeneity. Vermassen et al. (2015) used CE to resolve structural features and derive a urinary glycoprofile marker. Jia et al. (2017) analyzed RapiFluor-MS–labeled PSA glycans by HILIC-UPLC with fluorescence quantification and ESI-QTOF-MS for structural confirmation, whereas Nyalwidhe et al. (2013) examined permethylated glycans by MALDI-TOF/TOF with orthogonal validation by 2-AB labeling, normal-phase HPLC, exoglycosidase digestion, and lectin binding. Sample volume is a practical constraint, especially for direct EPS and EPS-derived EV, making MS-based workflows attractive because they can handle low input while delivering detailed structural information. Tandem MS generally provides higher confidence in determining glycan composition and certain linear structural features. However, it is often insufficient for the unambiguous assignment of glycosidic linkages and detailed branching patterns. Lectin-based assays, which rely on the selective binding of proteins to specific carbohydrate motifs, can indicate the presence of particular glycan epitopes but do not yield precise information on linkage position, branching architecture, or anomeric configuration. Definitive structural characterization of glycans, particularly with respect to linkage position and stereochemistry, typically requires complementary analytical approaches such as nuclear magnetic resonance (NMR) spectroscopy, exoglycosidase digestion, or other targeted enzymatic strategies.

Although EPS-focused glycomics studies remain few, emerging evidence suggests consistent trends, particularly reduced core fucosylation in cancer compared with non-cancer samples, with further decrease in more advanced disease. These observations support the potential of glycan profiles as diagnostic and prognostic biomarkers, but broader validation is required, as cohort sizes to date range from about 10 to 96 participants. Research on *O*-glycosylation alterations in PCa remains particularly limited, despite their strong links to malignancy, likely owing to technical challenges and the lack of standardized protocols for *O*-glycan handling and analysis. Furthermore, the lack of a unified framework and variability in experimental design constrain cross-study comparability and reproducibility. Progress in PCa glycomics will require greater standardization of analytical workflows and reporting. By synthesizing available data, Table 2 highlights recurrent glycosylation patterns associated with PCa progression and aggressiveness, helping to distinguish methodological from biological variation and underscoring glycan profiling as a promising non-invasive tool to refine PCa risk stratification.

### Potential integration with other omics studies

System glycobiology frameworks combine genomics, transcriptomics, proteomics, metabolomics, and glycomics, improving predictive performance and biological interpretability and providing a natural context for EPS-based glycan biomarker development (Kunej, 2019). In PCa, multi-layer analyses that merge DNA methylation, transcriptomics, proteomics and glycomics have already shown improved stratification of indolent *versus* aggressive disease, underscoring the value of combined molecular readouts (Murphy et al., 2018).

EPS, a prostate-proximal liquid biopsy enriched in prostate proteins and EVs, is well-suited to capture tumor-linked glycoforms and glycogene signatures, yet most PCa studies still focus on serum or urine due to PSA-based screening. PCa shows coordinated changes in both glycan structures and protein expression, producing composite biomarkers that resist pre-analytical variation and improve diagnostic accuracy (Scott & Munkley, 2019). Glycan traits, reflecting glycosyltransferase and glycosidase activities, yield stable pathway-level indices such as core fucosylation or sialylation (Xu et al., 2024). PSA glycoform profiling outperforms total PSA for detection (Yoneyama et al., 2014), while variation in glycogenes like MGAT3, MGAT5, FUT8, ST6GAL1, and ST3GAL1, and risk variants affecting PSA glycosylation, help explain inter-patient differences in EPS glyco-signatures (Barfeld et al., 2014; Srinivasan et al., 2019).

The metabolome and glycome are closely interconnected because pathways such as the HBP generate key donors like UDP-GlcNAc and feed sialic acid and fucose biosynthesis, thereby coupling carbohydrate and amino-sugar metabolism to *N-*glycosylation, *O-*glycosylation, and *O-*GlcNAcylation (Pham et al., 2017; Skurska & Olczak, 2024). These interdependencies motivate integrative analyses that relate EPS glycan features, particularly sialylation and core fucosylation indices, to metabolite signatures reflecting HBP activity and broader metabolic reprogramming in PCa (An et al., 2009; Gomez-Cebrian et al., 2019). As multi-omics integration frameworks mature and large consortia datasets such as TCGA and CPTAC become more widely used, EPS-based glycomics can be incorporated alongside genomic, transcriptomic, proteomic, and clinical variables to define molecular subgroups and to develop composite biomarker panels that more accurately reflect tumor biology than any single layer alone (Taylor et al., 2010; Subramanian et al., 2020).

### Challenges

Liquid biopsy offers clear advantages for cancer diagnosis and monitoring, but substantial technical, analytical, and clinical challenges still limit its routine implementation, and these apply equally to EPS-based assays (Pandey & Yadav, 2025). For regulatory approval and broad adoption, biomarkers require rigorous analytical validation, demonstration of reproducible clinical utility in large, prospective cohorts, and evidence that they improve decision-making, such as reducing unnecessary biopsies without missing clinically significant PCa or enhancing risk stratification beyond PSA and MRI, while remaining cost-effective and operationally feasible (Boukovala, Westphalen & Probst, 2024).

MS-based glycomics faces additional hurdles, including incomplete resolution of isomeric glycans without orthogonal separations or advanced fragmentation, matrix effects and ionization biases that favor relative rather than absolute quantification, and data analysis pipelines that remain more complex than standard proteomics (Pu et al., 2016; De Haan et al., 2022). Robust absolute quantification generally requires isotopically labeled standards and stringent quality control, which are not yet routine in most laboratories (Peng et al., 2022). In EPS-focused studies, pre-analytical variability in DRE technique, urine volume and dilution, timing, and sample handling can markedly affect the fraction of prostate-derived material, confounding biomarker signals unless carefully standardized and documented (Jiang et al., 2024).

**Figure 3:**
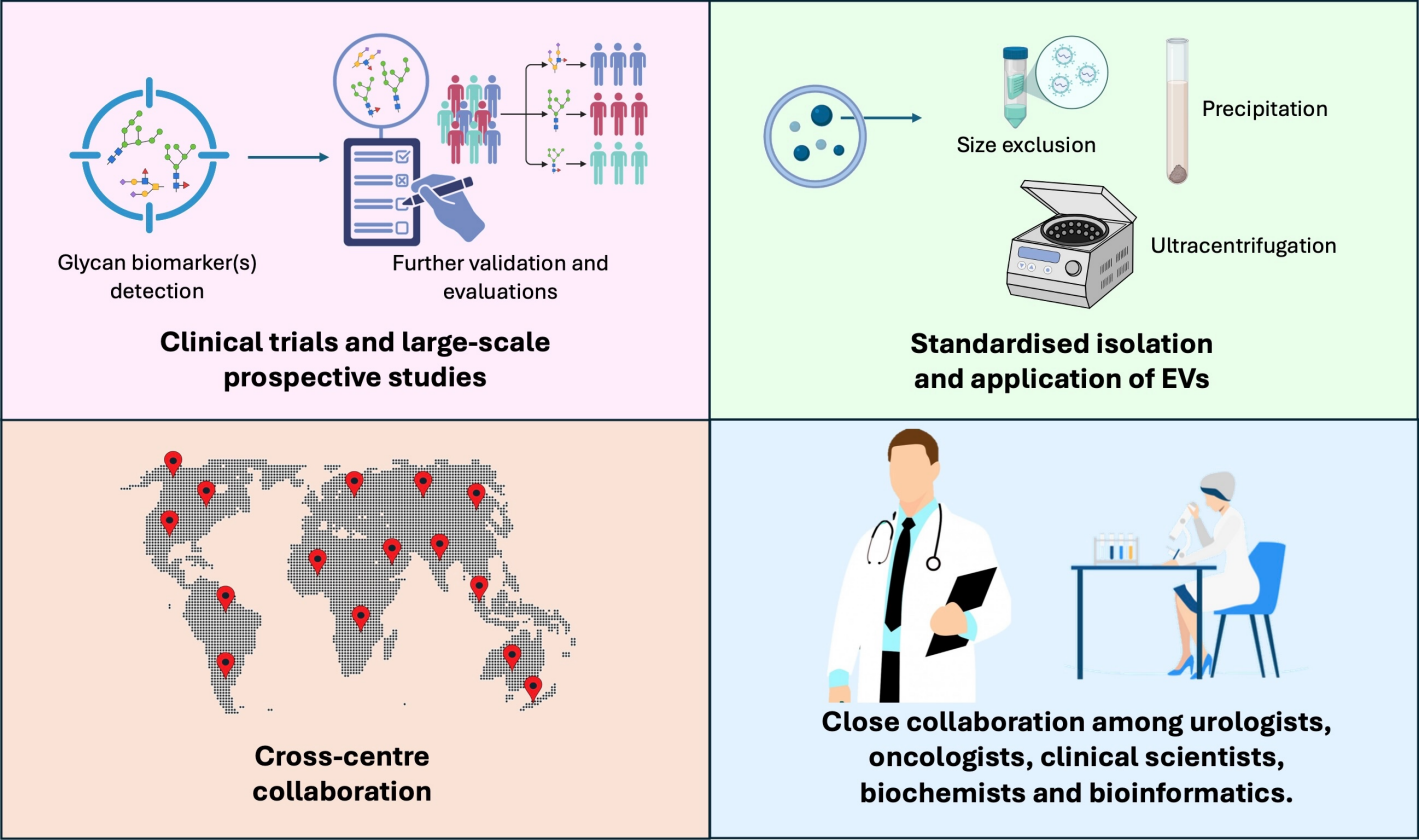
Translational roadmap for EPS glycan biomarkers. Key components required to translate laboratory-identified glycan features into clinically deployable assays, including pre-analytical standardization, EV isolation and quality control, clinically anchored study design, and prospective evaluation of clinical utility.

Using EVs as diagnostic tools introduces further complexity, including a lack of universally accepted, reproducible protocols for EV isolation and characterization, and the need for specialized, sometimes costly platforms for downstream analysis and multi-omics data integration (Ignatiadis, Sledge & Jeffrey, 2021; De Sousa et al., 2023). Most EPS glycan biomarker studies to date are observational and underpowered, suitable for discovery but insufficient to establish clinical utility. Interventional, biomarker-guided trials with pre-specific thresholds and clinically meaningful endpoints are needed to demonstrate patient benefit and clarify how EPS-derived readouts should inform clinical management (Boukovala, Westphalen & Probst, 2024). Addressing these challenges through pre-analytical standardization, harmonized EV and glycomics workflows, and carefully designed prospective studies will be essential before EPS-based glycomic biomarkers can be integrated into routine PCa care. Figure 3 illustrates key translational priorities, including pre-analytical standardization, EV isolation harmonization, analytical validation, and clinically anchored study design.

## Conclusion

Current PCa management relies on PSA-based testing, imaging and histopathology, but these tools do not fully meet the need for minimally invasive biomarkers that support early detection, refined risk stratifications, and longitudinal monitoring. EPS is a promising organ-proximal liquid biopsy enriched in prostate-derived proteins, metabolites, and EVs, yet its clinical use remains limited by pre-analytical variability, urine dilution, and a lack of standardized collection and processing protocols. Available studies consistently describe PCa-associated changes in core fucosylation, sialylation, and *N-*glycan branching in EPS-urine, direct EPS and EPS-derived EVs, with distinct patterns for bulk glycomes, and PSA glycoforms that likely reflect selective vesicle cargo packaging and diverse glycoprotein sources. Further progress will require improved methods for glycan isomer resolution and quantification, harmonized EV isolation and glycomics workflows, and large prospective studies that demonstrate added value beyond PSA and MRI in defined clinical decision pathways. Integrating EPS glycomics with proteomics and metabolomics, particularly readouts linked to nucleotide-sugar metabolism and the HBP, may further enhance biomarker performance and ultimately support risk-adapted screening, dynamic treatment monitoring, and earlier detection of therapeutic resistance for more precise, less invasive PCa management.

## Additional Information and Declarations

### Data Availability

The following information was supplied regarding data availability: This is a Literature Review.

## References

[ref-1] Ahmed HU, El-Shater Bosaily A, Brown LC, Gabe R, Kaplan R, Parmar MK, Collaco-Moraes Y, Ward K, Hindley RG, Freeman A, Kirkham AP, Oldroyd R, Parker C, Emberton M, Promis study group (2017). Diagnostic accuracy of multi-parametric MRI and TRUS biopsy in prostate cancer (PROMIS): a paired validating confirmatory study. Lancet.

[ref-2] An HJ, Kronewitter SR, De Leoz ML, Lebrilla CB (2009). Glycomics and disease markers. Current Opinion in Chemical Biology.

[ref-3] Ansari FJ, Tafti HA, Amanzadeh A, Rabbani S, Shokrgozar MA, Heidari R, Behroozi J, Eyni H, Uversky VN, Ghanbari H (2024). Comparison of the efficiency of ultrafiltration, precipitation, and ultracentrifugation methods for exosome isolation. Biochemistry and Biophysics Reports.

[ref-4] Attard G, Murphy L, Clarke NW, Sachdeva A, Jones C, Hoyle A, Cross W, Jones RJ, Parker CC, Gillessen S, Cook A, Brawley C, Gilson C, Rush H, Abdel-Aty H, Amos CL, Murphy C, Chowdhury S, Malik Z, Russell JM, Parkar N, Pugh C, Diaz-Montana C, Pezaro C, Grant W, Saxby H, Pedley I, O’Sullivan JM, Birtle A, Gale J, Srihari N, Thomas C, Tanguay J, Wagstaff J, Das P, Gray E, Alzouebi M, Parikh O, Robinson A, Montazeri AH, Wylie J, Zarkar A, Cathomas R, Brown MD, Jain Y, Dearnaley DP, Mason MD, Gilbert D, Langley RE, Millman R, Matheson D, Sydes MR, Brown LC, Parmar MKB, James ND, investigators S (2023). Abiraterone acetate plus prednisolone with or without enzalutamide for patients with metastatic prostate cancer starting androgen deprivation therapy: final results from two randomised phase 3 trials of the STAMPEDE platform protocol. The Lancet Oncology.

[ref-5] Barfeld SJ, East P, Zuber V, Mills IG (2014). Meta-analysis of prostate cancer gene expression data identifies a novel discriminatory signature enriched for glycosylating enzymes. BMC Medical Genomics.

[ref-6] Bastian K, Scott E, Elliott DJ, Munkley J (2021). FUT8 alpha-(1, 6)-fucosyltransferase in cancer. International Journal of Molecular Sciences.

[ref-7] Bennett AR, Lundstrom J, Chatterjee S, Thaysen-Andersen M, Bojar D (2025). Compositional data analysis enables statistical rigor in comparative glycomics. Nature Communications.

[ref-8] Blaschke CRK, Hartig JP, Grimsley G, Liu L, Semmes OJ, Wu JD, Ippolito JE, Hughes-Halbert C, Nyalwidhe JO, Drake RR (2021). Direct N-glycosylation profiling of urine and prostatic fluid glycoproteins and extracellular vesicles. Frontiers in Chemistry.

[ref-9] Boukovala M, Westphalen CB, Probst V (2024). Liquid biopsy into the clinics: current evidence and future perspectives. Journal of Liquid Biopsy.

[ref-10] Bray F, Laversanne M, Sung H, Ferlay J, Siegel RL, Soerjomataram I, Jemal A (2024). Global cancer statistics 2022: GLOBOCAN estimates of incidence and mortality worldwide for 36 cancers in 185 countries. CA: A Cancer Journal for Clinicians.

[ref-11] Brennan K, Martin K, FitzGerald SP, O’Sullivan J, Wu Y, Blanco A, Richardson C, Mc Gee MM (2020). A comparison of methods for the isolation and separation of extracellular vesicles from protein and lipid particles in human serum. Scientific Reports.

[ref-12] Buszewska-Forajta M, Monedeiro F, Golebiowski A, Adamczyk P, Buszewski B (2022). Citric acid as a potential prostate cancer biomarker determined in various biological samples. Metabolites.

[ref-13] Campbell MP, Peterson R, Mariethoz J, Gasteiger E, Akune Y, Aoki-Kinoshita KF, Lisacek F, Packer NH (2014). UniCarbKB: building a knowledge platform for glycoproteomics. Nucleic Acids Research.

[ref-14] Ceroni A, Maass K, Geyer H, Geyer R, Dell A, Haslam SM (2008). GlycoWorkbench: a tool for the computer-assisted annotation of mass spectra of glycans. Journal of Proteome Research.

[ref-15] Chung Y, Hong SK (2025). Evaluating prostate cancer diagnostic methods: the role and relevance of digital rectal examination in modern era. Investigative and Clinical Urology.

[ref-16] Cooper CA, Gasteiger E, Packer NH (2001). GlycoMod—a software tool for determining glycosylation compositions from mass spectrometric data. Proteomics.

[ref-17] Correll VL, Otto JJ, Risi CM, Main BP, Boutros PC, Kislinger T, Galkin VE, Nyalwidhe JO, Semmes OJ, Yang L (2022). Optimization of small extracellular vesicle isolation from expressed prostatic secretions in urine for in-depth proteomic analysis. Journal of Extracellular Vesicles.

[ref-18] Coughlan C, Bruce KD, Burgy O, Boyd TD, Michel CR, Garcia-Perez JE, Adame V, Anton P, Bettcher BM, Chial HJ, Konigshoff M, Hsieh EWY, Graner M, Potter H (2020). Exosome isolation by ultracentrifugation and precipitation and techniques for downstream analyses. Current Protocols in Cell Biology.

[ref-19] Dang XTT, Kavishka JM, Zhang DX, Pirisinu M, Le MTN (2020). Extracellular vesicles as an efficient and versatile system for drug delivery. Cell.

[ref-20] De Haan N, Narimatsu Y, Koed Moller Aasted M, Larsen ISB, Marinova IN, Dabelsteen S, Vakhrushev SY, Wandall HH (2022). In-depth profiling of O-glycan isomers in human cells using C18 nanoliquid chromatography-mass spectrometry and glycogenomics. Analytical Chemistry.

[ref-21] De Sousa KP, Rossi I, Abdullahi M, Ramirez MI, Stratton D, Inal JM (2023). Isolation and characterization of extracellular vesicles and future directions in diagnosis and therapy. Wiley Interdisciplinary Reviews: Nanomedicine and Nanobiotechnology.

[ref-22] De Vos II, Luiting HB, Roobol MJ (2023). Active surveillance for prostate cancer: past, current, and future trends. Journal of Personalized Medicine.

[ref-23] Delafield DG, Li L (2021). Recent advances in analytical approaches for glycan and glycopeptide quantitation. Molecular & Cellular Proteomics.

[ref-24] Diaz-Fernandez A, Ryo Jochumsen M, Christensen NL, Dalsgaard Sorensen K, Bouchelouche K, Borre M, Holm Vendelbo M, Ferapontova EE (2024). Liquid-biopsy glycan score biomarker accurately indicates and stratifies primary and metastatic prostate cancers. Analytical Chemistry.

[ref-25] Ding N, Nie H, Sun X, Sun W, Qu Y, Liu X, Yao Y, Liang X, Chen CC, Li Y (2011). Human serum N-glycan profiles are age and sex dependent. Age and Ageing.

[ref-26] Drago D, Andolfo A, Mosca E, Orro A, Nocera L, Cucchiara V, Bellone M, Montorsi F, Briganti A (2021). A novel expressed prostatic secretion (EPS)-urine metabolomic signature for the diagnosis of clinically significant prostate cancer. Cancer Biology & Medicine.

[ref-27] Drake RR, Elschenbroich S, Lopez-Perez O, Kim Y, Ignatchenko V, Ignatchenko A, Nyalwidhe JO, Basu G, Wilkins CE, Gjurich B, Lance RS, Semmes OJ, Medin JA, Kislinger T (2010). In-depth proteomic analyses of direct expressed prostatic secretions. Journal of Proteome Research.

[ref-28] Drake RR, White KY, Fuller TW, Igwe E, Clements MA, Nyalwidhe JO, Given RW, Lance RS, Semmes OJ (2009). Clinical collection and protein properties of expressed prostatic secretions as a source for biomarkers of prostatic disease. Journal of Proteomics.

[ref-29] Erdbrugger U, Blijdorp CJ, Bijnsdorp IV, Borras FE, Burger D, Bussolati B, Byrd JB, Clayton A, Dear JW, Falcon-Perez JM, Grange C, Hill AF, Holthofer H, Hoorn EJ, Jenster G, Jimenez CR, Junker K, Klein J, Knepper MA, Koritzinsky EH, Luther JM, Lenassi M, Leivo J, Mertens I, Musante L, Oeyen E, Puhka M, Van Royen ME, Sanchez C, Soekmadji C, Thongboonkerd V, Van Steijn V, Verhaegh G, Webber JP, Witwer K, Yuen PST, Zheng L, Llorente A, Martens-Uzunova ES (2021). Urinary extracellular vesicles: a position paper by the Urine Task Force of the International Society for extracellular vesicles. Journal of Extracellular Vesicles.

[ref-30] Freitas D, Balmana M, Pocas J, Campos D, Osorio H, Konstantinidi A, Vakhrushev SY, Magalhaes A, Reis CA (2019). Different isolation approaches lead to diverse glycosylated extracellular vesicle populations. Journal of Extracellular Vesicles.

[ref-31] Garnham R, Geh D, Nelson R, Ramon-Gil E, Wilson L, Schmidt EN, Walker L, Adamson B, Buskin A, Hepburn AC, Hodgson K, Kendall H, Frame FM, Maitland N, Coffey K, Strand DW, Robson CN, Elliott DJ, Heer R, Macauley M, Munkley J, Gaughan L, Leslie J, Scott E (2024). ST3 beta-galactoside alpha-2, 3-sialyltransferase 1 (ST3Gal1) synthesis of Siglec ligands mediates anti-tumour immunity in prostate cancer. Communications Biology.

[ref-32] Gebrehiwot AG, Melka DS, Kassaye YM, Rehan IF, Rangappa S, Hinou H, Kamiyama T, Nishimura SI (2018). Healthy human serum N-glycan profiling reveals the influence of ethnic variation on the identified cancer-relevant glycan biomarkers. PLOS ONE.

[ref-33] Gomez-Cebrian N, Rojas-Benedicto A, Albors-Vaquer A, Lopez-Guerrero JA, Pineda-Lucena A, Puchades-Carrasco L (2019). Metabolomics contributions to the discovery of prostate cancer biomarkers. Metabolites.

[ref-34] Graham SM, Krieger JN, Githua PL, Wamuyu LW, Wale S, Ramko KM, Dragavon JA, Muller CH, Holte SE, Mandaliya KN, McClelland RS, Peshu NM, Sanders EJ, Coombs RW (2011). Post-prostatic massage fluid/urine as an alternative to semen for studying male genitourinary HIV-1 shedding. Sexually Transmitted Infections.

[ref-35] Gratacos-Mulleras A, Duran A, Asadi Shehni A, Ferrer-Batalle M, Ramirez M, Comet J, De Llorens R, Saldova R, Llop E, Peracaula R (2020). Characterisation of the main PSA glycoforms in aggressive prostate cancer. Scientific Reports.

[ref-36] Hamid Y, Rabbani RD, Afsara R, Nowrin S, Ghose A, Papadopoulos V, Sirlantzis K, Ovsepian SV, Boussios S (2025). Exosomal liquid biopsy in prostate cancer: a systematic review of biomarkers for diagnosis, prognosis, and treatment response. International Journal of Molecular Sciences.

[ref-37] He M, Zhou X, Wang X (2024). Glycosylation: mechanisms, biological functions and clinical implications. Signal Transduction and Targeted Therapy.

[ref-38] Heidenreich A, Aus G, Bolla M, Joniau S, Matveev VB, Schmid HP, Zattoni F, European Association of U (2008). EAU guidelines on prostate cancer. European Urology.

[ref-39] Ignatiadis M, Sledge GW, Jeffrey SS (2021). Liquid biopsy enters the clinic—implementation issues and future challenges. Nature Reviews Clinical Oncology.

[ref-40] Jayaprakash NG, Surolia A (2017). Role of glycosylation in nucleating protein folding and stability. Biochemical Journal.

[ref-41] Jia G, Dong Z, Sun C, Wen F, Wang H, Guo H, Gao X, Xu C, Xu C, Yang C, Sun Y (2017). Alterations in expressed prostate secretion-urine PSA N-glycosylation discriminate prostate cancer from benign prostate hyperplasia. Oncotarget.

[ref-42] Jiang S, Lu F, Chen J, Jiao Y, Qiu Q, Nian X, Qu M, Wang Y, Li M, Liu F, Gao X (2024). UPCARE: urinary extracellular vesicles-derived prostate cancer assessment for risk evaluation. Journal of Extracellular Vesicles.

[ref-43] Kalluri R, LeBleu VS (2020). The biology, function, and biomedical applications of exosomes. Science.

[ref-44] Khoo A, Liu LY, Nyalwidhe JO, Semmes OJ, Vesprini D, Downes MR, Boutros PC, Liu SK, Kislinger T (2021). Proteomic discovery of non-invasive biomarkers of localized prostate cancer using mass spectrometry. Nature Reviews Urology.

[ref-45] Kizuka Y, Taniguchi N (2016). Enzymes for N-glycan branching and their genetic and nongenetic regulation in cancer. Biomolecules.

[ref-46] Kumar BS (2024). Recent developments and application of mass spectrometry imaging in N-glycosylation studies: an overview. Mass Spectrometry.

[ref-47] Kunej T (2019). Rise of systems glycobiology and personalized glycomedicine: why and how to integrate glycomics with multiomics science?. OMICS.

[ref-48] Larssen P, Wik L, Czarnewski P, Eldh M, Lof L, Ronquist KG, Dubois L, Freyhult E, Gallant CJ, Oelrich J, Larsson A, Ronquist G, Villablanca EJ, Landegren U, Gabrielsson S, Kamali-Moghaddam M (2017). Tracing cellular origin of human exosomes using multiplex proximity extension assays. Molecular & Cellular Proteomics.

[ref-49] Laubli H, Borsig L (2019). Altered cell adhesion and glycosylation promote cancer immune suppression and metastasis. Frontiers in Immunology.

[ref-50] Li P, Wang L, Guo R, Feng H, Ji Y, Lim SY, Ng BH, Laserna AKC, Khan S, Chen SM, Li SFY (2022). Cross-identification of N-glycans by CE-LIF using two capillary coatings and three labeling dyes. Talanta.

[ref-51] Lim J, Malek R, Jr S, Toh CC, Sundram M, Woo SYY, Yusoff NAM, Teh GC, Chui BJT, Ngu IS, Thevarajah S, Koh WJ, Lee SB, Khoo SC, Teoh BW, Zainal R, Tham TM, Omar S, Nasuha NA, Akaza H, Ong TA, Study MC (2021). Prostate cancer in multi-ethnic Asian men: real-world experience in the Malaysia Prostate Cancer (M-CaP) study. Cancer Medicine.

[ref-52] Lucena MC, Carvalho-Cruz P, Donadio JL, Oliveira IA, De Queiroz RM, Marinho-Carvalho MM, Sola-Penna M, De Paula IF, Gondim KC, McComb ME, Costello CE, Whelan SA, Todeschini AR, Dias WB (2016). Epithelial mesenchymal transition induces aberrant glycosylation through hexosamine biosynthetic pathway activation. Journal of Biological Chemistry.

[ref-53] Mechref Y, Hu Y, Desantos-Garcia JL, Hussein A, Tang H (2013). Quantitative glycomics strategies. Molecular & Cellular Proteomics.

[ref-54] Mereiter S, Balmana M, Campos D, Gomes J, Reis CA (2019). Glycosylation in the era of cancer-targeted therapy: where are we heading?. Cancer Cell.

[ref-55] Merriel SWD, Pocock L, Gilbert E, Creavin S, Walter FM, Spencer A, Hamilton W (2022). Systematic review and meta-analysis of the diagnostic accuracy of prostate-specific antigen (PSA) for the detection of prostate cancer in symptomatic patients. BMC Medicine.

[ref-56] Messina A, Palmigiano A, Esposito F, Fiumara A, Bordugo A, Barone R, Sturiale L, Jaeken J, Garozzo D (2021). HILIC-UPLC-MS for high throughput and isomeric N-glycan separation and characterization in Congenital Disorders Glycosylation and human diseases. Glycoconjugate Journal.

[ref-57] Micanovic R, LaFavers K, Garimella PS, Wu XR, El-Achkar TM (2020). Uromodulin (Tamm-Horsfall protein): guardian of urinary and systemic homeostasis. Nephrology, Dialysis, Transplantation.

[ref-58] Molnarova K, Cokrtova K, Tomnikova A, Krizek T, Kozlik P (2022). Liquid chromatography and capillary electrophoresis in glycomic and glycoproteomic analysis. Monatshefte für Chemie.

[ref-59] Murphy K, Murphy BT, Boyce S, Flynn L, Gilgunn S, O’Rourke CJ, Rooney C, Stockmann H, Walsh AL, Finn S, O’Kennedy RJ, O’Leary J, Pennington SR, Perry AS, Rudd PM, Saldova R, Sheils O, Shields DC, Watson RW (2018). Integrating biomarkers across omic platforms: an approach to improve stratification of patients with indolent and aggressive prostate cancer. Molecular Oncology.

[ref-60] National Cancer Registry (2023). National cancer registry report 2017–2021.

[ref-61] Neelamegham S, Aoki-Kinoshita K, Bolton E, Frank M, Lisacek F, Lutteke T, O’Boyle N, Packer NH, Stanley P, Toukach P, Varki A, Woods RJ, Group SD (2019). Updates to the symbol nomenclature for glycans guidelines. Glycobiology.

[ref-62] Nolan JP, Duggan E (2018). Analysis of individual extracellular vesicles by flow cytometry. Methods in Molecular Biology.

[ref-63] Nyalwidhe JO, Betesh LR, Powers TW, Jones EE, White KY, Burch TC, Brooks J, Watson MT, Lance RS, Troyer DA, Semmes OJ, Mehta A, Drake RR (2013). Increased bisecting N-acetylglucosamine and decreased branched chain glycans of N-linked glycoproteins in expressed prostatic secretions associated with prostate cancer progression. Proteomics Clinical Applications.

[ref-64] Pandey S, Yadav P (2025). Liquid biopsy in cancer management: integrating diagnostics and clinical applications. Practical Laboratory Medicine.

[ref-65] Pasala C, Sharma S, Roychowdhury T, Moroni E, Colombo G, Chiosis G (2024). N-glycosylation as a modulator of protein conformation and assembly in disease. Biomolecules.

[ref-66] Peng W, Kobeissy F, Mondello S, Barsa C, Mechref Y (2022). MS-based glycomics: an analytical tool to assess nervous system diseases. Frontiers in Neuroscience.

[ref-67] Pham ND, Pang PC, Krishnamurthy S, Wands AM, Grassi P, Dell A, Haslam SM, Kohler JJ (2017). Effects of altered sialic acid biosynthesis on N-linked glycan branching and cell surface interactions. Journal of Biological Chemistry.

[ref-68] Pietrobono S, Stecca B (2021). Aberrant sialylation in cancer: biomarker and potential target for therapeutic intervention?. Cancer.

[ref-69] Pinto D, Parameswaran R (2023). Role of truncated O-GalNAc glycans in cancer progression and metastasis in endocrine cancers. Cancer.

[ref-70] Pu Y, Ridgeway ME, Glaskin RS, Park MA, Costello CE, Lin C (2016). Separation and identification of isomeric glycans by selected accumulation-trapped ion mobility spectrometry-electron activated dissociation tandem mass spectrometry. Analytical Chemistry.

[ref-71] Ramirez-Garrastacho M, Bajo-Santos C, Line A, Martens-Uzunova ES, De la Fuente JM, Moros M, Soekmadji C, Tasken KA, Llorente A (2022). Extracellular vesicles as a source of prostate cancer biomarkers in liquid biopsies: a decade of research. British Journal of Cancer.

[ref-72] Riley NM, Malaker SA, Driessen MD, Bertozzi CR (2020). Optimal dissociation methods differ for N- and O-glycopeptides. Journal of Proteome Research.

[ref-73] Roberts MJ, Richards RS, Chow CWK, Buck M, Yaxley J, Lavin MF, Schirra HJ, Gardiner RA (2017). Seminal plasma enables selection and monitoring of active surveillance candidates using nuclear magnetic resonance-based metabolomics: a preliminary investigation. Prostate International.

[ref-74] Royo F, Zuniga-Garcia P, Sanchez-Mosquera P, Egia A, Perez A, Loizaga A, Arceo R, Lacasa I, Rabade A, Arrieta E, Bilbao R, Unda M, Carracedo A, Falcon-Perez JM (2016). Different EV enrichment methods suitable for clinical settings yield different subpopulations of urinary extracellular vesicles from human samples. Journal of Extracellular Vesicles.

[ref-75] Schoberer J, Shin YJ, Vavra U, Veit C, Strasser R (2018). Analysis of protein glycosylation in the ER. Methods in Molecular Biology.

[ref-76] Scott E, Archer Goode E, Garnham R, Hodgson K, Orozco-Moreno M, Turner H, Livermore K, Putri Nangkana K, Frame FM, Bermudez A, Jose Garcia Marques F, McClurg UL, Wilson L, Thomas H, Buskin A, Hepburn A, Duxfield A, Bastian K, Pye H, Arredondo HM, Hysenaj G, Heavey S, Stopka-Farooqui U, Haider A, Freeman A, Singh S, Johnston EW, Punwani S, Knight B, McCullagh P, McGrath J, Crundwell M, Harries L, Heer R, Maitland NJ, Whitaker H, Pitteri S, Troyer DA, Wang N, Elliott DJ, Drake RR, Munkley J (2023). ST6GAL1-mediated aberrant sialylation promotes prostate cancer progression. Journal of Pathology.

[ref-77] Scott E, Munkley J (2019). Glycans as biomarkers in prostate cancer. International Journal of Molecular Sciences.

[ref-78] Sekhoacha M, Riet K, Motloung P, Gumenku L, Adegoke A, Mashele S (2022). Prostate cancer review: genetics, diagnosis, treatment options, and alternative approaches. Molecules.

[ref-79] Serkova NJ, Gamito EJ, Jones RH, O’Donnell C, Brown JL, Green S, Sullivan H, Hedlund T, Crawford ED (2008). The metabolites citrate, myo-inositol, and spermine are potential age-independent markers of prostate cancer in human expressed prostatic secretions. Prostate.

[ref-80] Shao H, Im H, Castro CM, Breakefield X, Weissleder R, Lee H (2018). New technologies for analysis of extracellular vesicles. Chemical Reviews.

[ref-81] Shrivastava P, Garg H, Bhat M, Dinda A, Kumar R (2020). Urinary prostate-specific antigen and microseminoprotein-beta levels in men with and without prostate cancer: a prospective cohort study. Indian Journal of Urology.

[ref-82] Skurska E, Olczak M (2024). Interplay between *de novo* and salvage pathways of GDP-fucose synthesis. PLOS ONE.

[ref-83] Slomka A, Wang B, Mocan T, Horhat A, Willms AG, Schmidt-Wolf IGH, Strassburg CP, Gonzalez-Carmona MA, Lukacs-Kornek V, Kornek MT (2022). Extracellular vesicles and circulating tumour cells—complementary liquid biopsies or standalone concepts?. Theranostics.

[ref-84] Smith BAH, Bertozzi CR (2021). The clinical impact of glycobiology: targeting selectins, Siglecs and mammalian glycans. Nature Reviews Drug Discovery.

[ref-85] Smith SF, Brewer DS, Hurst R, Cooper CS (2024). Applications of urinary extracellular vesicles in the diagnosis and active surveillance of prostate cancer. Cancer.

[ref-86] Srinivasan S, Stephens C, Wilson E, Panchadsaram J, De Voss K, Koistinen H, Stenman UH, Brook MN, Buckle AM, Klein RJ, Lilja H, Clements J, Batra J, Practical C (2019). Prostate cancer risk-associated single-nucleotide polymorphism affects prostate-specific antigen glycosylation and its function. Clinical Chemistry.

[ref-87] Subramanian I, Verma S, Kumar S, Jere A, Anamika K (2020). Multi-omics data integration, interpretation, and its application. Bioinformatics and Biology Insights.

[ref-88] Tajiri M, Ohyama C, Wada Y (2008). Oligosaccharide profiles of the prostate specific antigen in free and complexed forms from the prostate cancer patient serum and in seminal plasma: a glycopeptide approach. Glycobiology.

[ref-89] Taniguchi N, Ohkawa Y, Maeda K, Harada Y, Nagae M, Kizuka Y, Ihara H, Ikeda Y (2021). True significance of N-acetylglucosaminyltransferases GnT-III, V and alpha1, 6 fucosyltransferase in epithelial-mesenchymal transition and cancer. Molecular Aspects of Medicine.

[ref-90] Tapper W, Carneiro G, Mikropoulos C, Thomas SA, Evans PM, Boussios S (2024). The application of radiomics and AI to molecular imaging for prostate cancer. Journal of Personalized Medicine.

[ref-91] Taylor BS, Schultz N, Hieronymus H, Gopalan A, Xiao Y, Carver BS, Arora VK, Kaushik P, Cerami E, Reva B, Antipin Y, Mitsiades N, Landers T, Dolgalev I, Major JE, Wilson M, Socci ND, Lash AE, Heguy A, Eastham JA, Scher HI, Reuter VE, Scardino PT, Sander C, Sawyers CL, Gerald WL (2010). Integrative genomic profiling of human prostate cancer. Cancer Cell.

[ref-92] Tsechelidis I, Vrachimis A (2022). PSMA PET in imaging prostate cancer. Frontiers in Oncology.

[ref-93] Urabe F, Yamada Y, Yamamoto S, Tsuzuki S, Kimura S, Ochiya T, Kimura T (2024). Extracellular vesicles and prostate cancer management: a narrative review. Translational Andrology and Urology.

[ref-94] Van de Wall S, Santegoets KCM, Van Houtum EJH, Bull C, Adema GJ (2020). Sialoglycans and Siglecs can shape the tumor immune microenvironment. Trends in Immunology.

[ref-95] Varki A, Cummings RD, Esko JD, Stanley P, Hart GW, Aebi M, Mohnen D, Kinoshita T, Packer NH, Prestegard JH, Schnaar RL, Seeberger PH (2022). Essentials of glycobiology.

[ref-96] Velonas VM, Woo HH, Dos Remedios CG, Assinder SJ (2013). Current status of biomarkers for prostate cancer. International Journal of Molecular Sciences.

[ref-97] Vermassen T, Van Praet C, Lumen N, Decaestecker K, Vanderschaeghe D, Callewaert N, Villeirs G, Hoebeke P, Van Belle S, Rottey S, Delanghe J (2015). Urinary prostate protein glycosylation profiling as a diagnostic biomarker for prostate cancer. Prostate.

[ref-98] Wang S, Kozarek J, Russell R, Drescher M, Khan A, Kundra V, Barry KH, Naslund M, Siddiqui MM (2024). Diagnostic performance of prostate-specific antigen density for detecting clinically significant prostate cancer in the era of magnetic resonance imaging: a systematic review and meta-analysis. European Urology Oncology.

[ref-99] Wang M, Zhu J, Lubman DM, Gao C (2019). Aberrant glycosylation and cancer biomarker discovery: a promising and thorny journey. Clinical Chemistry and Laboratory Medicine.

[ref-100] Wilkinson H, Saldova R (2020). Current methods for the characterization of O-glycans. Journal of Proteome Research.

[ref-101] Xu X, Barreiro K, Musante L, Kretz O, Lin H, Zou H, Huber TB, Holthofer H (2019). Management of Tamm-Horsfall protein for reliable urinary analytics. Proteomics Clinical Applications.

[ref-102] Xu X, Peng Q, Jiang X, Tan S, Yang W, Han Y, Oyang L, Lin J, Shen M, Wang J, Li H, Xia L, Peng M, Wu N, Tang Y, Wang H, Liao Q, Zhou Y (2024). Altered glycosylation in cancer: molecular functions and therapeutic potential. Cancer Communications.

[ref-103] Yamamoto M, Harada Y, Suzuki T, Fukushige T, Yamakuchi M, Kanekura T, Dohmae N, Hori K, Maruyama I (2019). Application of high-mannose-type glycan-specific lectin from Oscillatoria Agardhii for affinity isolation of tumor-derived extracellular vesicles. Analytical Biochemistry.

[ref-104] Yoneyama T, Ohyama C, Hatakeyama S, Narita S, Habuchi T, Koie T, Mori K, Hidari KI, Yamaguchi M, Suzuki T, Tobisawa Y (2014). Measurement of aberrant glycosylation of prostate specific antigen can improve specificity in early detection of prostate cancer. Biochemical and Biophysical Research Communications.

[ref-105] Yu H, Wang J, Tang Z, Li X, Yin M, Zhang F, Shu J, Chen W, Yang S, Li Z (2020). Integrated glycomics strategy for the evaluation of glycosylation alterations in salivary proteins associated with type 2 diabetes mellitus. RSC Advances.

[ref-106] Zhang Y, Sun L, Lei C, Li W, Han J, Zhang J, Zhang Y (2022). A sweet warning: mucin-type O-glycans in cancer. Cell.

[ref-107] Zijlstra C, Stoorvogel W (2016). Prostasomes as a source of diagnostic biomarkers for prostate cancer. Journal of Clinical Investigation.

